# In Situ Filler Addition for Homogeneous Dispersion of Carbon Nanotubes in Multi Jet Fusion–Printed Elastomer Composites

**DOI:** 10.1002/advs.202300593

**Published:** 2023-07-03

**Authors:** Jiayao Chen, Ran An, Wei Shian Tey, Qingyun Zeng, Lihua Zhao, Kun Zhou

**Affiliations:** ^1^ HP‐NTU Digital Manufacturing Corporate Lab School of Mechanical and Aerospace Engineering Nanyang Technological University Singapore 639798 Singapore; ^2^ Singapore Centre for 3D Printing School of Mechanical and Aerospace Engineering Nanyang Technological University Singapore 639798 Singapore; ^3^ 3D Lab HP Labs HP Inc. Palo Alto CA 94304 USA

**Keywords:** 3D printing, dual‐functional toughness agents, in situ filler addition, site‐specific mechanical properties

## Abstract

The dispersibility of fillers determines their effect on the mechanical properties and anisotropy of the 3D‐printed polymeric composites. Nanoscale fillers have the tendency to aggregate, resulting in the deterioration of part performance. An in situ filler addition method using the newly developed dual‐functional toughness agents (TAs) is proposed in this work for the homogeneous dispersion of carbon nanotubes (CNTs) in elastomer composites printed via multi jet fusion. The CNTs added in the TAs serve as an infrared absorbing colorant for selective powder fusion, as well as the strengthening and toughening fillers. The printability of the TA is theoretically deduced based on the measured physical properties, which are subsequently verified experimentally. The printing parameters and agent formulation are optimized to maximize the mechanical performance of the printed parts. The printed elastomer parts show significant improvement in strength and toughness for all printing orientations and alleviation of the mechanical anisotropy originating from the layer‐wise fabrication manner. This in situ filler addition method using tailorable TAs is applicable for fabricating parts with site‐specific mechanical properties and is promising in assisting the scalable manufacturing of 3D‐printed elastomers.

## Graphical abstract

The in situ filler addition method applying dual‐functional toughness agents is developed for the 3D printing of composite elastomers with homogeneous filler dispersion and isotopically enhanced mechanical properties. By tailoring the local compositions with different agents, the parts with tunable mechanical properties are built to showcase the capability in achieving modulated deformations.

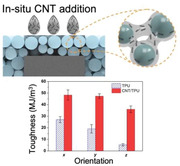

## 1 Introduction

The addition of reinforcement materials to polymers is a typical way to improve their mechanical performance for applications in load bearing, safety and protections, friction and wear, etc.^[^ ^1^ ^]^ The production of polymer composites, initially achieved through conventional processing, has expanded to the emerging additive manufacturing (also known as 3D printing) techniques due to their ability to customize production with great design freedom.^[^ ^2^ ^]^ In the fabrication process, the preparation of composite feedstocks is particularly important, as the dispersibility, stability, and compatibility of fillers in polymer matrices determine the processability of the feedstocks and the properties of the final parts. There are primarily two methods to physically introduce fillers to a 3D‐printed object.^[^ ^3^ ^]^ One method is to premix fillers with the polymer matrix prior to printing, and the other is to add the fillers to the matrix in situ.

The premixing method with feedstocks that can be readily used for printing is the predominant production method for most 3D printing techniques, including powder bed fusion (PBF), fused filament fabrication (FFF), direct ink writing, and vat photopolymerization techniques.^[^ ^3^, ^4^ ^]^ PBF, in particular, is the technique utilizing a heat source to coalesce powder particles in a powder bed to build parts with excellent mechanical properties and complex geometries without additional supporting structures.^[^ ^5^ ^]^ Many kinds of premixed composite powders with reinforcements such as glass fibers, carbon fibers, and aramid fibers have been developed for PBF.^[^ ^6^ ^]^ The alignment of the fibers generated by the moving roller along the recoating direction induces the increase in strength and modulus of the fiber‐reinforced composites, but inevitably causes an increase in brittleness and anisotropy.^[^ ^7^ ^]^ Mechanical properties of the parts in directions other than the fiber alignment direction are poor, particularly in the building direction due to insufficient interlayer adhesion. To alleviate the increased brittleness and anisotropy, nanofillers such as carbon nanotubes (CNTs) and graphene are chosen as fillers for PBF because of their isotropic reinforcement determined by the nanoscale size.^[^ ^8^ ^]^ Currently, wet mixing, a premix method for preparing composite powder, is the main way to obtain nanoparticle‐filled composite powder for PBF.^[^ ^4a^ ^]^ Nanofillers are coated onto the surfaces of insoluble polymer powder though intermolecular interactions in the presence of a solvent.^[^ ^4a^ ^]^ Nevertheless, a large portion of these nanocomposite parts, such as CNT/thermoplastic polyurethane (TPU) and graphene/TPU, exhibited decreased mechanical properties compared with those of their neat polymer counterparts,^[^ ^9^ ^]^ commonly because of nanoparticle agglomeration. Efficiently enhancing the dispersion of nanofillers and improving the mechanical properties of the printed parts remains a challenge for PBF. In addition, the premixing of feedstocks prohibits the variation of local filler concentration and the addition of other types of fillers, as the material composition is fixed.

In contrast, the in situ addition of fillers during printing offers greater design freedom by enabling the selection of material compositions and customization of site‐specific properties of the printed parts. For example, the in situ fabrication of composite filaments for the FFF technique is a representative method where a tow of continuous fibers is in situ embedded into the thermoplastics on demand to achieve local mechanical enhancement.^[^ ^10^ ^]^ The proper in situ addition of fillers is a promising composite production method as it provides effective reinforcement, allows for locally varied properties, reduces material wastage, and is free from tedious feedstock preparation. Till now, there are barely any studies on the development of in situ prepared composites for 3D printing techniques except FFF.

Multi jet fusion (MJF), combining PBF with the thermal inkjet printing technology, additively manufactures parts by fusing and coalescing powder selectively at regions where a black fusing agent (FA) is dispensed for absorbing infrared energy. A detailing agent mainly containing water is applied to the perimeter of the patterns to obtain parts with high dimensional accuracy and fine detail. Recently, we reported the MJF 3D voxel printing process for building voxelated conductive elastomers using the multifunctional agent.^[^ ^11^ ^]^ The voxel printing method, although initially proposed for the site‐specific variation of electrical properties, showed its potential in realizing the in situ addition of nanoscale reinforcements into a printed part in a programmable manner. Thus, to fully utilized the in situ reinforcement addition in MJF, new functional agents need to be developed for efficient mechanical enhancement and high tunability of site‐specific mechanical properties in the printed parts.

Here, we develop a series of CNT‐containing toughening agents (TAs) that can be homogenously dispensed onto the polymer powders during MJF printing, allowing for on‐demand adjustments of the printing process. Through the in situ addition of CNTs to the TPU parts, the TA serves dual functionalities as both an infrared‐absorbing colorant and an agent for effective strengthening and toughening, achieving in situ addition of CNTs in TPU parts. The CNT/TPU parts printed with TA exhibited significant increases in toughness and strength, and substantial alleviation of the mechanical anisotropy was observed. The agent formulation and printing process were systematically varied to optimize the performance of the printed parts. The dispersion of the CNTs during the printing process was investigated to reveal the underlying mechanism of the performance enhancement. Furthermore, by tailoring the local composition with different TAs, the parts with tunable mechanical properties were fabricated to showcase the capability of achieving modulated deformation. This work provides an efficient way to print reinforced nanocomposite parts with in situ addition of nanofillers, which is also promising in the manufacturing of functional parts with site‐specific mechanical properties.

## 2 Results and Discussion

### 2.1 MJF Printing Process with In Situ Addition of CNTs

**Figure 1:**
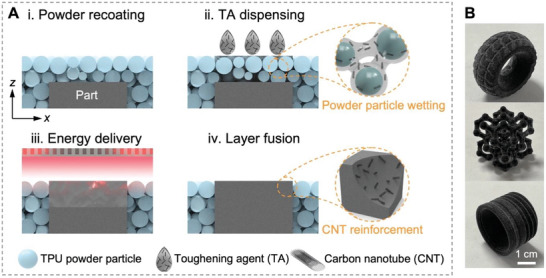
The TA‐assisted MJF printing process with in situ CNT addition. A) Schematic of the printing process comprising of four stages: i) powder recoating, ii) TA dispensing, iii) energy delivery, and iv) layer fusion. B) Photos of parts printed via this printing process.

The printing process of the in situ CNTs addition to a TPU matrix using the TA on a customized MJF testbed is shown in **Figure** 1A. A layer of TPU powder is recoated onto the print bed by a roller moving from the supply bed to the print bed in the *y* direction. The mean particle size of the TPU powder is ≈70–90 µm,^[^ ^12^ ^]^ and the printing layer thickness was set at 80 µm, indicating the covering of one single powder layer on the print bed upon each recoating. Subsequently, a carriage packed with thermal inkjet printheads scans across the print bed in the *x* direction to dispense the TA onto the designated regions. TA droplets loaded with CNTs contact and promptly wet the surface of the powder particles. After the evaporation of the water in the droplets, the CNTs are homogeneously coated on the surfaces of the powder particles. An infrared energy source mounted on the carriage passes over the print bed, and the dark CNT colorant absorbs sufficient heat, enabling the powder particles within the selected regions to melt. It is worth noting that the TA is completely capable of replacing the original fusing agent (FA, mainly containing carbon black, a commercial fusing agent for MJF's Jet Fusion printing systems)^[^ ^13^ ^]^ in this work. The printheads can be programmed to dispense varied quantities of TAs in the desired regions through the activation of multiple ink cartridges. The process is repeated until a part has been formed completely. The CNTs are dispersed and immobilized by the fused TPU matrix after cooling of the print bed. Figure 1B shows some intricate 3D parts printed via this printing process. As the CNTs in the TA are only deposited on the region where the powder particles are meant to fuse and consolidate into a final part, the loose TPU powder (without CNT addition) can be completely recycled by mixing with fresh powder at a certain ratio.

**Figure 2:**
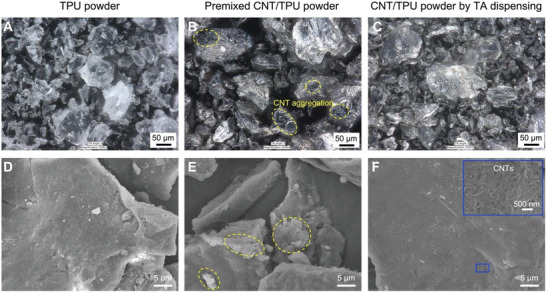
Morphology of the TPU and CNT/TPU powder. A,D) TPU powder, B,E) premixed CNT/TPU powder fabricated by wet mixing, and C,F) CNT/TPU powder fabricated by TA dispensing.

The dispersibility of nanofillers in a polymer matrix determines the mechanical performance of the composites. Uniform distribution of the nanofiller results in performance enhancement, while the aggregation of the nanofillers causes local stress concentration and consequently poor mechanical properties. In this work, the nanofillers were homogeneously dispersed in the TA and precisely dispensed as 20 µm droplets onto the TPU powder bed at the desired locations, ensuring the dispersion of CNTs in the TPU matrix. The morphology of the TPU powder bed before droplet dispensing is presented in **Figures** **2**A,D. To verify the CNT dispersion on the TPU powder particles by this proposed in situ filler addition method using the TA, the images of the powder bed after droplet dispensing were compared with those of the premixed powder by common wet mixing. The CNT addition amount was validated to be comparable through thermogravimetric analysis (TGA, Figure S1, Supporting Information). For the wet mixing of the nanofillers and powders, the CNT re‐aggregation occurred during the powder drying process due to the high specific surface area and surface energy,^[^ ^4a^ ^]^ as shown in Figures 2B,E. On the contrary, the droplet dispensing system enables the addition of nanofillers to the powder bed through the individual micro‐scale droplets with extremely short evaporation time, making the aggregation of nanofillers near impossible. In addition, the system is programmed to deposit droplets with precise control over the amount and location (dispensing resolution shown in Figure S2, Supporting Information), resulting in high homogeneity of a supper thin CNT coating on each polymer powder particle. In Figures 2C,F, the homogeneous CNT dispersion on the surfaces of the powder particles is illustrated, suggesting higher efficiency of CNT reinforcement than the premixed method. The microscope images in larger scales are shown in Figure S3, Supporting Information to further confirm the CNT dispersity. Moreover, the processing challenges arising from the poor powder flowability of the premixed composite powder^[^ ^9a^ ^]^ can be feasibly resolved through the in situ addition method.

### 2.2 Agent Design and Printability

CNTs have exceptional strength, modulus, and toughness, which endow CNTs and their composites with a number of exciting potential applications in aerospace, automobile, electronics, energy, etc.^[^ ^1a^ ^]^ The CNTs in TA perform dual functionalities of both energy absorption and mechanical enhancement. The selection of hydrophilic carboxylated CNTs served the purpose of enhancing their hydrogen‐bonding interaction with TPU molecular chains, as well as achieving a stable suspension in the liquid solution consisting of a mixture of water and 2‐pyrrolidinone. Water can be superheated by a thin film resistor located within the ink channel of the thermal inkjet printheads, which generates vapor bubbles to eject a droplet. Due to its low surface tension and high boiling point, 2‐pyrrolidinone was included to reduce the surface tension and evaporation rate of the agent to enable powder particle wetting and prevent nozzle clogging. To replace the FA in MJF printing, stable jetting in the thermal inkjet system and sufficient heat absorption for the fusion of powder particles are two major requirements for the TA.

**Figure 3:**
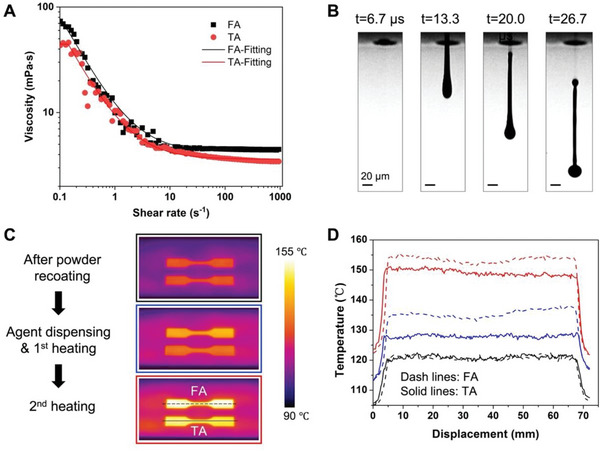
Printability of the TA. A) The logarithm of viscosity versus the logarithm of shear rates for the TA and FA. The solid lines correspond to the best fit of the data to the Carreau model. B) Droplet formation for the TA. C) Temperature mapping of the print bed building CNT/TPU and FA/TPU tensile bars at three stages including after powder recoating, after agent dispensing and with the fusing lamps turned on, and after the return pass of the carriage with second round heating. D) The corresponding temperature distribution along the center lines of the two tensile bars showed in (C) at the three stages.

To investigate the jetting performance of the TAs in the thermal inkjet system, one representative TA, containing 3 wt% CNTs and 13 wt% 2‐pyrrolidinone, was selected to characterize its physical properties, which includes the density *ρ*, surface tension *γ*, and rheological properties. **Figure** **3**A displays the rheological properties of the TA and FA, and the TA has a lower viscosity than the FA at high shear rates. Both agents exhibited shear‐thinning behavior, and the viscosity *η* decreases remarkably with an increasing shear rate and reaches a plateau termed infinite shear viscosity. The extrapolated values of the infinite shear viscosity from model fitting were given using the Carreau model^[^ ^14^ ^]^ and used for the following calculation because the droplet generation was under an ultrahigh shear rate process.^[^ ^15^ ^]^ Based on these measured properties, the inverse Ohnesorge number, *Z* (*Z* = $\sqrt{\gamma \rho a}/$ *η*), for a droplet generated from a nozzle with a diameter of *a* can be calculated (Table S1, Supporting Information). The values of *Z* for the TA and FA were 7.9 and 5.1, respectively, both within the jettable range between 1 and 14, indicating the stable droplet jetting of TA and FA.^[^ ^16^ ^]^ To validate the jettability of TA, a series of the TA droplets formation from the thermal inkjet nozzle was recorded by a high‐speed camera, as shown in Figure 3B. A liquid column which was pushed out of the nozzle eventually detached from the printhead and broke into a main droplet with a diameter of 20 µm and a few small satellites. The train of the ejected TA droplets was not deflected, and CNT agglomeration on the nozzle plate during firing was not observed, suggesting the stable jetting of the TA droplets.

To explore the energy absorption of TA, the surface temperature of the powder bed was measured during the printing process. Figures 3C,D display the temperature mapping of the print bed during the printing of tensile bars with TA and FA. After recoating a new layer of TPU powder, the surface temperature of the regions on top of the previous fusing layer was around 120 °C. As the carriage passed over the print bed, the printheads dispensed TA and FA in the designated regions, respectively, and both fusing lamps mounted on the carriage were turned on. The temperature of the powder dispensed with the FA rapidly raised to 135–140 °C, higher than that of the region dispensed with the TA (125–130 °C), suggesting that the radiation absorbing efficiency of the FA was higher than that of the TA at the beginning. After the return pass of the carriage which provided a second round of irradiation exposure, both regions were further heated to 145–155 °C, reaching the melting temperature of TPU (Figure S4A, Supporting Information). It can be concluded that this newly invented TA possesses sufficient energy absorption for the MJF printing process. The developed TA shows decent printability and meets the requirements for the printing process.

### 2.3 Mechanical Enhancement of the Printed Parts

Due to the desired printability of the TA and the capability of homogenous CNT dispersion, the MJF printing with in situ filler addition method is expected to achieve significant mechanical enhancement in the printed parts. To obtain optimal mechanical performance, the effects of the TA formulation and printing parameters on the mechanical properties of the printed parts were investigated with regard to CNT content, solvent content and type, agent dispensing volume, layer thickness, and build orientation. The tensile properties of all printed parts are listed in Table S2, Supporting Information.

**Figure 4:**
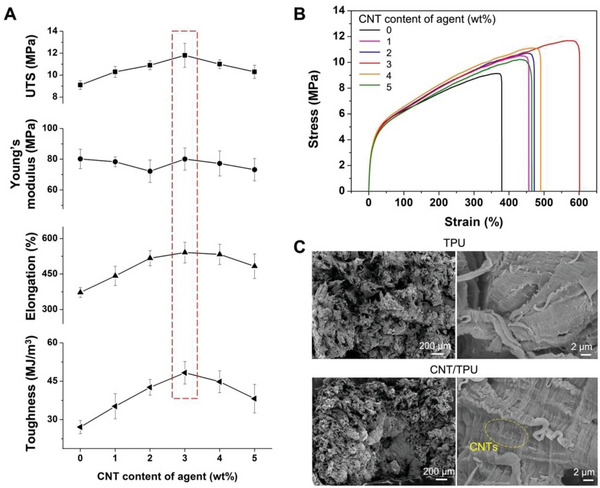
Effect of CNT content of the agent on the mechanical properties of the printed parts. Comparison of A) tensile properties and B) Stress–strain curves of the TPU and CNT/TPU parts fabricated using FA (denoted by 0 wt% CNT) and TAs with CNT concentration ranging from 1 to 5 wt%. C) Fractographs of the TPU fabricated with FA containing carbon black (upper panel) and the CNT/TPU parts fabricated with TA with 3 wt% CNTs (bottom panel), respectively.

**Figures** **4**A,B show the tensile properties and stress‐strain curves measured in the *x* direction for the TPU parts fabricated using FA and the CNT/TPU parts fabricated using TAs with CNT content from 1 to 5 wt%. The results show that the UTS, elongation at break, and toughness of the parts first increased and then decreased with the increase in CNT content, suggesting an optimal CNT content of 3 wt% when using TA. Based on the equations listed in Experimental Procedures, the mass fractions of the CNTs in the parts fabricated using TAs with 1, 2, 3, 4, and 5 wt% CNTs were approximately 0.06, 0.12, 0.18, 0.24, and 0.30 wt%, respectively. Although CNTs can reinforce printed parts, excessive CNTs may tend to aggregate and interrupt the continuity of the polymer matrices. Consequentially, the mechanical properties of the parts may degrade, which is a common issue in many composite manufacturing processes. The UTS, elongation at break, and toughness of the CNT/TPU part with the optimal mechanical properties were increased by 30% (11.8 MPa), 45% (541%), and 78% (48.3 MJ m^−3^), respectively, compared with the neat TPU parts fabricated using FA. The Young's modulus of the parts resulted in no significant changes due to the combined influence of the CNTs and solvent. The density result showed insignificant variation among the printed parts (Figure S5, Supporting Information). Figure 4C shows the fractographs of the TPU and the CNT/TPU tensile bars. Both fracture surfaces were irregular and rough and exhibited some dimpling, illustrating a ductile fracture process in which cavities and fibrils were formed. The fatigue striations were visible in the high‐magnification images, representing individual crack‐growth steps. The fractured CNT/TPU tensile bars had a smaller cross‐sectional area than that of the TPU counterparts, demonstrating large amounts of plastic buckling and deformation.

The effects of solvent content and solvent type on mechanical performance were explored and presented. As the content of 2‐pyrroidinone in a printable agent falls within the range of 10–25 wt% for MJF printing according to a patent from HP,^[^ ^17^ ^]^ TAs with low (13 wt%) or high (20 wt%) 2‐pyrrolidinone content were fabricated to investigate the effect of the solvent content on the tensile properties of the printed parts (Table S2, Supporting Information). The mechanical properties exhibited insignificant variations between the parts with different solvent content, suggesting the ineffectiveness of the solvent in enhancing the performance of the final part. However, the properties of the solvent may affect the wetting of the powder in the printing process and the properties of the printed part. To investigate this effect, another type of solvent, glycerol, with higher surface tension (62.0 mN m^−1^), was used to replace the 2‐pyrrolidinone (surface tension 32.4 mN m^−1^) in TA. The result of the tensile test revealed reductions in both the UTS and the toughness of the parts printed with glycerol, confirming the role of solvents on the performance of the printed part. It should be noted that solvents with high boiling points in the FA and TA remain in the final printed parts at TPU printing condition and act as plasticizers. The agent compositions affect the mechanical properties, thermal properties, and rheological behavior as shown in Figures S4B and S6, Supporting Information. Due to the larger amount of carbon fillers in FA, the TPU parts fabricated with FA exhibited a higher glass transition temperature (hard domains), molten viscosity, and elastic modulus than those of the CNT/TPU parts fabricated with TA. The molten viscosity of the CNT/TPU parts underwent an initial increase and a subsequent decrease at a range of low frequency, possibly because of the good interfacial bonding between the CNTs and the matrix against the molecule flow.

In addition to the TA formulation, the printing parameters, including the TA dispensing times and layer thickness, were changed to explore their effects on the mechanical performance of the printed parts. The representative TA, containing 3 wt% CNTs and 13 wt% 2‐pyrrolidinone, was set to dispense twice to change the dispense volume of TA during printing. In the experiment, the TA was dispensed in both the forward and return passes of the carriage to achieve twice dispensing. However, as shown in Table S2, Supporting Information, the mechanical properties of the parts with twice TA dispensing decreased as compared with those of the parts with single TA dispensing. It could be attributed to the insufficient fusion caused by the increased heat loss due to twice the amount of the solvent plasticizer and water evaporation. Hence, there is a correlation balance between the increase in CNT content and the decrease in fusing quality. Three distinct layer thicknesses, 60, 80, and 100 µm were chosen to test their effect on the performance of the printed part when the representative TA was dispensed once. Generally, the result showed insignificant changes in the tensile properties with building layer thicknesses of 60 and 80 µm. However, the parts with a building layer thickness of 100 µm exhibited slightly lower mechanical properties than those of other layer thicknesses, possibly attributed to the lower amount of CNTs per unit volume. In conclusion, the optimal combination of agent formula and printing parameter is determined to be a layer thickness of 80 µm and one‐time dispensing of TA with 3 wt% CNT and 13 wt% 2‐pyrrolidinone.

**Figure 5:**
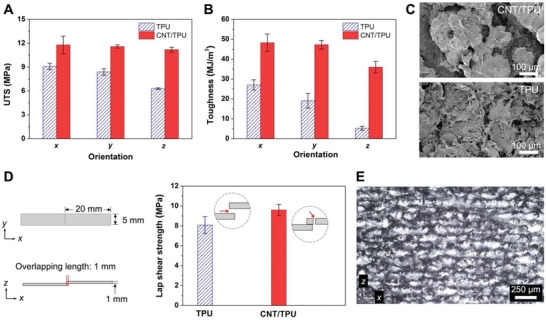
Effect of the build orientation on the tensile properties of the neat TPU and CNT/TPU parts. A) The UTS and B) toughness of the parts measured along the *x*, *y*, and *z* directions. C) Fractographs of the neat TPU and CNT/TPU parts fractured under tensile loading along the *z* direction. D) Specimen size of the parts with an overlapping area of 5 mm × 1 mm for the lap shear test (left panel), the lap shear strength of the neat TPU and CNT/TPU parts (right panel). The insets show the fracture modes of the corresponding parts. E) Optical image of a cross‐sectional ultrathin slice cut from the CNT/TPU part. The bright and dark regions refer to TPU and CNTs, respectively.

Using the optimized parameters, the effect of build orientation on the mechanical properties of the parts was investigated. As shown in **Figures** **5**A,B, the UTS and toughness of the CNT/TPU parts were significantly improved in the *x*, *y*, and particularly the *z* directions. It is worth noting that the UTS in the *z* direction (11.2 MPa) was close to those in the *x* (11.8 MPa) and *y* directions (11.6 MPa), indicating significant alleviation of the mechanical anisotropy resulting from PBF. The average increments of the UTS and toughness of the CNT/TPU parts were 46% and 155%, compared with those of the neat TPU parts (Table S3, Supporting Information). Figure 5C shows the fractographs of the neat TPU and CNT/TPU parts fractured under tensile loading along the *z* direction. Many fibrillar structures can be observed in the fracture surface of the CNT/TPU part, while the fracture surface of the TPU part is relatively smooth, indicating that the CNT/TPU parts underwent stronger strain hardening than the neat TPU parts. Since the TPU powder has a narrow sintering window (4.8 °C, Figure S4A, Supporting Information), crystallization of the molten TPU may occur before a new layer is fused, resulting in weak interlayer bonding and significant mechanical anisotropy. The lap shear test was conducted to further confirm the strengthening effect of the TA on interlayer bonding (Figure 5D). The lap shear strength was calculated by dividing the maximum load by the overlapping area. The CNT/TPU parts fabricated using TA exhibited a higher lap shear strength (9.6 MPa) than that of the neat TPU counterparts (8.1 MPa). In addition, different fracture modes were observed between the two parts. The upper and bottom layers in the overlapped area were separated from each other for the neat TPU parts, whereas either one of the layers was fractured in the CNT/TPU parts, indicating an improved interlayer bonding induced by the TA.

Such significant mechanical enhancement in the CNT/TPU parts can be attributed to the homogenous dispersion of CNTs with the in situ addition method, as well as the functionalized surface of the CNTs, where the carboxyl groups bond with the polar groups of the TPU molecular chains via hydrogen‐bonding interaction. To quantitatively investigate the variation of hydrogen bonds between CNT/TPU and TPU parts, the Fourier transform infrared spectra were analyzed (Figures S7, S8 and Table S4, Supporting Information). The hydrogen‐bonding interaction increased with the CNT addition, according to the increased quantity of hydrogen‐bonded N—H and C=O groups.^[^ ^18^ ^]^ As a result, the TA serves as a glue to strengthen the interlayer adhesion and thus promotes excellent mechanical performance in the printed parts.

Cross‐sectional interlayer morphology of the CNT/TPU part was observed from an ultrathin slice cut along the *z* direction (Figure 5E). Since the TA was homogenously dispensed on the TPU powder particles, the CNTs were distributed mostly on the top surface of the powder particles and partially in the gaps between the particles, generating a CNT network across the polymer matrix as shown in the optical image. Due to the random emergence of the gaps between the particles, the network irregularly grew along the *z* direction. Such reinforcement network indicates a similar concept as the previously reported segregated architectures that were constructed by hot pressing nanofiller‐coated polymer granules.^[^ ^19^ ^]^ Aided by the drop‐on‐demand inkjet technology, this in situ filler addition method can be further developed to actualize the programmable formation of reinforcement networks in 3D printing.

To highlight the advantages of the in situ filler addition method using the newly invented TA in this work, the mechanical properties of the previously reported TPU‐based composite parts printed by another PBF technique, Selective Laser Sintering (SLS), are listed in **Table** **1**. Most of the reported SLS‐printed TPU composite parts exhibit a reduction in the mechanical properties, while the mechanical properties of the parts in this work were improved due to the effective CNT addition and strong interfacial adhesion between CNTs and TPU matrix, indicating the enhanced capability of the TA with the proposed method. In addition to PBF, other fabrication processes of the CNT/TPU composites reported in recent years mainly include extrusion‐based additive manufacturing (Fused Deposition Modeling, FDM) and conventional manufacturing such as casting.^[^ ^4^, ^20^ ^]^ The mechanical properties of these composites are also compared in Table S5, Supporting Information. It is known that the porosity of PBF‐printed parts is generally higher than that of the FDM‐printed parts (especially in the *x*–*y* plane) or injection molded parts, as the PBF‐printed parts are consolidated based on the fusion of numerous micro‐scale powder particles. Hence, although some of the CNT/TPU composites printed by FDM exhibit higher strength improvement than this work, their mechanical strengths increase with sacrificing extensibility. Moreover, in addition to the mechanical enhancement of TA, the CNT/TPU parts fabricated using TA are cost‐effective, and their consumable cost is comparable to that of the normal TPU fabricated using commercial FA (Table S6, Supporting Information). The benefit of CNT/TPU part manufacturing was further calculated as 63.6% using the reference of toughness enhancement, suggesting the high mechanical improvement efficiency and cost‐effectiveness of the proposed method.

**Table 1:** Comparison of mechanical properties of CNT‐filled and graphene‐filled TPU composites printed via SLS and MJF. Average values in the *x*, *y*, and *z* directions were used as the mechanical results of this work

Technique (filler addition method)	Part	Filler content [wt%]	UTS [MPa]	Variation^a)^ [%]	Elongation [%]	Variation^b)^ [%]	Ref.
SLS (Wet mixing)	MWCNT^c)^/TPU	0.5	4.8	−55.6	210	−51.7	[9a]
	SWCNT^d)^/TPU	0.05	20	−7.0	525	−5.4	[9b]
	MWCNT/TPU	0.2	17	−20.9	450	−18.9	[9b]
	Graphene/TPU	0.5	6.5	−69.8	290	−47.7	[9b]
	SWCNT/TPU	0.2	17.5	−25.5	465	−10.6	[9c]
	MWCNT/TPU	0.25	9.1	/	/	/	[21]
	Graphene/TPU	0.2	1.1	−76.1	50	−68.8	[22]
MJF (In situ addition)	MWCNT/TPU	0.18	11.5	+46	502	+100	This work

^a)^ Variation of the UTS relative to that of the neat TPU parts;

^b)^ Variation of the elongation relative to that of the neat TPU parts;

^c)^ MWCNT: multi‐walled carbon nanotubes;

^d)^ SWCNT: single‐walled carbon nanotubes.

### 2.4 Modulated Deformation of the CNT/TPU Parts

**Figure 6:**
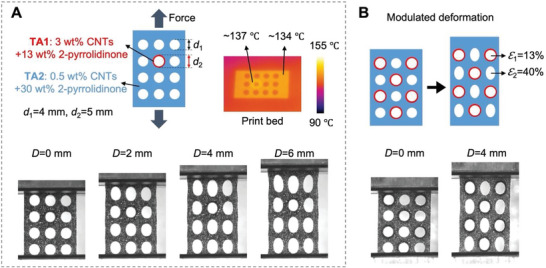
Modulated deformation of the CNT/TPU parts with programmable dispensing of two TAs. A) Design and deformation of the CNT/TPU part with an array of holes. The holes were designed with the same diameter of 4 mm but differed in the material around the hole. The annular region around one of the holes was printed using the TA1 (red circle) and the remaining region was applied with TA2 (topleft). Temperature mapping of the print bed in printing showed a slight difference in temperature between the two regions (topright). Different modes of deformation appeared for two types of holes under the displacement of 0, 2, 4, and 6 mm (bottom). B) Design and deformation of the CNT/TPU part with half of the holes strengthened using the TA1 (top). The deformation of the part under uniaxial stretching was schematically displayed and experimentally recorded for different deformation modes and elongation rates (bottom).

This in situ addition method can be further extended for locally varying the mechanical properties within one printed part by using TAs with different formulations through the drop‐on‐demand inkjet system. Following such a method, the degree of deformation of the printed parts was modulated using two types of TAs, and the outcomes are presented in **Figure** **6**. One TA with 3 wt% CNTs and 13 wt% 2‐pyrrolidinone was denoted as TA1, while the other one with 0.5 wt% CNTs and 30 wt% 2‐pyrrolidinone was labeled as TA2. The printed part using TA1 had better mechanical performance than that with TA2 (Table S2, Supporting Information). An array of circular through holes with a diameter of 4 mm was designed on the part, where one of the holes was reinforced by dispending TA1 once in the annular area of the hole, while in the remaining region, TA2 was dispensed twice (Figure 6A). In the printing process, the temperature mapping of the print bed shows a slight temperature difference between the reinforced annular area (≈137 °C) and the matrix region (≈134 °C), as a higher CNT content enables slightly stronger irradiance absorption. The infrared absorption is thus comparable for the two agents upon synchronous printing. Under uniaxial stretching, the circular holes of the part transformed into elliptical shapes except the one reinforced with TA1, indicating that deformation can be locally altered by this in situ addition method via changing the TA composition, dispensing position, and dispensing volume. Another design with a greater number of strengthened holes is presented in Figure 6B, illustrating distinct hole deformations with average elongations of 13% and 40% for the holes with and without reinforcement, respectively.

This in situ filler addition method is able to control the amount and location of the fillers within a single part using the drop‐on‐demand printheads. The method offers additional design freedom as opposed to composites fabricated using traditional manufacturing methods, where various components of composite feedstocks need to be prepared for fabricating parts with different filler amounts, and programmable filler distribution and site‐specific properties are not possible. Such a method using TAs can be extended to arbitrary 2D and 3D architectures. It is known that lattice architectures from structural design are capable of exhibiting a broader range of mechanical responses governed by their geometry. By combining exquisite structures with our method of tailoring constituent materials, more design freedom is attainable in fabricating functional materials or metamaterials with predetermined mechanical properties.

## 3 Conclusion

In conclusion, we present an effective in situ filler addition method by a new dual‐functional agent for MJF printing to enhance the mechanical performance and alleviate mechanical anisotropy for elastomers. The dual‐functional TA containing carboxylated CNTs served as both a colorant for infrared absorption and an agent for strengthening and toughening. The physical properties of the agent were measured to predict the printability of the agent which was experimentally verified by high‐speed imaging. With the aid of the drop‐on‐demand printheads, the CNTs were homogenously added to the TPU matrix via the dispensing of 20 µm TA droplets. The agent formulation and printing parameters were systematically varied to optimize the mechanical performance of the printed part. The optimal CNT/TPU parts showed substantial improvements in the averaged UTS and toughness by 46% (11.5 MPa) and 155% (43.9 MJ m^−3^), respectively. The mechanical enhancement in the *z* direction was significantly increased, which could be caused by the improved interfacial bonding resulting from the hydrogen‐bonding interaction formed between the polar groups from the carboxylated CNTs and TPU molecular chains. The mechanical increment of the printed parts presented in this study outperforms most of the reported CNT/TPU elastomers fabricated by other 3D printing techniques. To demonstrate the modulated deformation functionality, parts with programmable additive composition and mechanical properties were fabricated by applying TAs with different CNT content.

This facile and efficient in situ fabrication method avoids the tedious preparation process of composite feedstocks and the relevant compatibility and dispersibility issues of the powder. Moving forward, in situ polymerization and site‐specific crystallization are attainable through the development of agents containing cross‐linkers or nucleators, respectively. It is promising to promote the development of manufacturing 3D printed polymers and composites with excellent and tailorable mechanical properties for applications of soft robots, wearable protections, predetermined load‐bearing devices, information encryption, etc.

## 4 Experimental Section

### Fabrication of Toughening Agent

In this work, the TA containing 3 wt% CNTs produced optimal mechanical performance. 23 g carboxylated multi‐walled CNT water suspension (13 wt%, 10 µm in length, and > 50 nm in diameter, surfactant‐include, XFNANO Materials Tech Co. Ltd., China), 64 g deionized water, and 13 g 2‐pyrrolidinone (Sigma‐Aldrich Pty. Ltd., USA) were fully mixed with a probe sonicator (Q500, Qsonica L.L.C, USA) at 40% amplitude. To remove the small amount of inevitable CNT agglomeration, which might clog the nozzles of printheads during droplet jetting, the mixture was filtered with 1‐µm pore size nylon filters and sonicated for 45 min. Four rounds of filtering and ultrasonication were performed before filling the TA into an empty ink cartridge. The TAs with various CNT content (1–5 wt%), solvent type (glycerol, Sigma‐Aldrich Pty. Ltd., USA), and solvent content (10–20 wt%) were prepared following a similar procedure according to **Table** **2**. The premixed CNT/TPU powder was prepared by wet mixing as the control group according to the reference.^[^ ^9a^ ^]^

**Table 2:** Toughening agent formulation

Ingredient	Content [wt%]
Carboxylated multi‐walled CNTs	1–5
Non‐ionic surfactant	0–1
2‐pyrrolidinone or glycerol	10–20
Water	balanced

### Printing Parameters

TPU is one of the most widely used elastomer powders in PBF, and the toughness enhancement of TPU can further broaden their applications. Hence, TPU powder (Ultrasint TPU01, HP Inc., USA) was used for the demonstration of the in situ addition method. The CNT/TPU composite parts were printed using a customized MJF 3D printing testbed (HP Inc., USA) with TPU powder. Different from the MJF commercial model in which the printing process is fixed with each type of powder, this testbed is capable of realizing a tunable printing process on demand. Here, the printing process was adjusted to print the composite parts with in situ addition of CNTs, and relevant printing parameters including the thermal inkjet settings, energy input, the temperature of the print bed, layer thickness, recoating speed, supply ratio of powder, etc., were tuned as followings.

Before printing, TA was filled into an empty ink cartridge with a printhead nozzle diameter of 16 µm. The carriage scanning speed was set at 16 in. s^−1^. At this scanning speed and a droplet dispensing resolution of 600 dots per inch (DPI) in the *x* direction (carriage moving direction), the firing frequency of droplets was 9.6 kHz. The resolution of droplet dispensing in the *y* direction (roller recoating direction) was 1200 DPI. The volume of TA dispensed can be tailored by controlling the number of dispensing times. For example, twice TA dispensing volume can be obtained by dispensing TA in two separate passes or by using two active printheads within the same pass.

The heating system of the testbed is composed of an overhead lamp above the powder bed and two fusing lamps on both sides of the moving carriage. The building layer thickness was set between 60 and 100 µm, and the roller speed was set at 250 mm s^−1^. The supply ratio, which is defined as the ratio of the distance moved by the rising supply bed to that of the sinking print bed, was fixed at 1.8 to obtain a dense powder layer. By adjusting the power of the overhead lamp, the fusing lamps, and the temperature controlling system around the print bed, the regions of TPU powder dispensed with TA or FA were heated to 130–150 °C for TPU coalescence to occur. The layerwise dispensing of TA and powder recoating process was repeated until the final parts were completed. The parts were collected and cleaned by bead blasting after the printing.

### Calculation of CNT Mass Fraction

The mass fractions *φ* of CNTs in a CNT/TPU part can be calculated by

(1) $$\varphi \;=\frac{m_{c}}{m_{p}}\;$$

The mass of CNTs in the part, *m*_c_ can be expressed as

(2) $$m_{c}=\;AD_{x}D_{y}m_{d}pwn$$

where *A* is the cross‐sectional area of the printed part; *D_x_* and *D_y_* are the resolution of droplet dispensing in the *x* and *y* directions, respectively; *m*_d_ is the mass of a droplet (12 ng for the nozzle diameter of 16 µm); *p* is the percentage of the nozzles activated for the print job (40%); *w* is the CNT mass fraction in the TA (1–5 wt%); *n* is the number of building layers.

The mass of the printed part *m*_p_ can be calculated by

(3) $$m_{p}=\;\rho Anh$$

where *ρ* is the density of the TPU part (*ρ* = 1.1 g cm^−3^), and *h* is the thickness of a building layer.

### Characterization

The CNT loading fractions were measured by a TGA instrument (TGA‐Q500, TA Instruments, USA) with a ramp rate of 10 °C min^−1^ under a nitrogen atmosphere. The sintering window and thermal properties of the TPU powder and printed parts were evaluated by a differential scanning calorimetry system (DSC‐Q200, TA Instruments, USA) with a ramp rate of 10 °C min^−1^. The temperature distribution of the powder bed was recorded by an infrared camera (A655sc, FLIR Systems Inc., USA). The apparent viscosity of the agents was measured at shear rates ramping from 0.01 to 1000 s^–1^ by a rotational rheometer (DHR‐2, TA Instruments, USA) with a parallel plate geometry of 40 mm in diameter. The melt viscosity of the printed parts was measured at shear rates ramping from 0.001 to 1000 s^–1^ under the melting temperature of 150 °C, and the frequency sweep of the melted samples was also conducted from 0.1 to 100 rad s^−1^. The surface tension and contact angle of the agents were obtained by an optical tensiometer (Attension Theta Flex, Biolin Scientific, Finland). The powder morphology was captured by a digital microscope (VHX‐7100, Keyence, Japan). The jettability of TA was tested using HP internal testing system with a high‐speed camera (Fast Cam SA‐X2, Photron, Japan) for microscopic droplet imaging. The voltage, firing frequency, and pulse width were set at 29 V, 20 kHz, and 1.2, respectively.

The tensile properties of the printed parts were evaluated by a universal tester (AGX 10 kN, Shimadzu Corp., Japan) in accordance with the ASTM D638 Type V standard (length gauge × width × thickness: 9.53 mm × 3.18 mm × 3.18 mm) at a test speed of 10 mm min^−1^. Four specimens were tested for each group. If there is no specific description, the parts were printed with a TA consisting of 3 wt% CNT and 13 wt% 2‐pyrrolidinone, a building layer thickness of 80 µm, and one‐time TA dispensing. The density of the printed parts was measured by an analytical balance (XS204, Mettler Toledo, USA). The fractographs of the CNT/TPU parts were observed by a scanning electron microscope (JSM‐5600 LV, JEOL, Japan). The densities of the printed parts were measured by an analytical balance (XS204, Mettler Toledo, USA). FTIR spectra were measured by the IR Prestige 21 (Shimadzu, Japan).

## Conflict of Interest

The authors declare no conflict of interest.

## Supporting information

Supporting Information

## Data Availability

The data that support the findings of this study are available in the supplementary material of this article.
