# Supplementary material for: In Situ Filler Addition for Homogeneous Dispersion of Carbon Nanotubes in Multi Jet Fusion–Printed Elastomer Composites
Source: Adv Sci (Weinh). 2023 Jul 3;10(25):2300593. doi: 10.1002/advs.202300593 (PMC10477867; doi:10.1002/advs.202300593)
Supplement: Supplementary file 1 — Supporting Information [file ADVS-10-2300593-s001.pdf]

ADVANCED SCIENCE
Open Access

Supporting Information

for *Adv. Sci.*, DOI 10.1002/advs.202300593

In Situ Filler Addition for Homogeneous Dispersion of Carbon Nanotubes in Multi Jet Fusion–Printed Elastomer Composites

*Jiayao Chen, Ran An, Wei Shian Tey, Qingyun Zeng, Lihua Zhao and Kun Zhou**

Supporting Information

In-situ filler addition for homogeneous dispersion of carbon nanotubes in Multi Jet Fusion–printed elastomer composites

Jiayao Chen,[a,b,†] *Ran An,*[a,b,†] *Wei Shian Tey,*[a,b] *Qingyun Zeng,*[a] *Lihua Zhao,*[a,c] *and Kun Zhou*[a,b,*]

The carbon nanotube (CNT) loading fractions of the premixed CNT/thermoplastic polyurethane (TPU) powder and the CNT/TPU part fabricated using one-time dispensing of the toughness agent (TA) containing 3 wt% CNTs and 13 wt% 2-pyrrolidinone were investigated by thermogravimetric analysis (TGA). As shown in Figure S1, the weight loss percentage was almost identical between the two samples, indicating same loading fraction of CNTs between the premixed and in-situ addition methods.

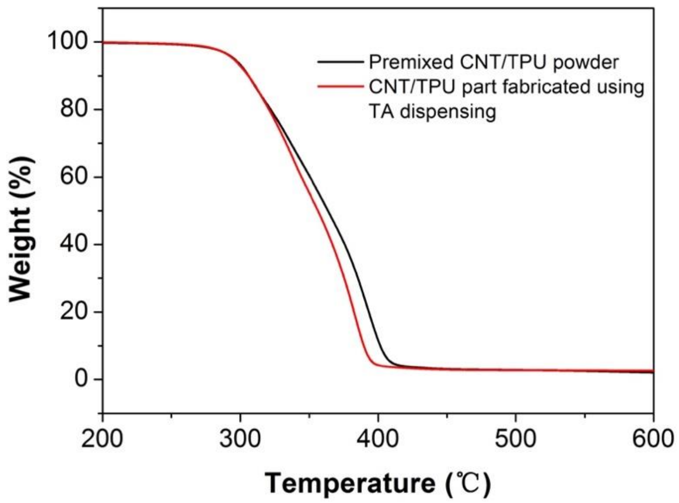

Figure S1. TGA curves of the premixed CNT/TPU powder and the CNT/TPU part fabricated using TA dispensing. The latter was calibrated by removing a proportion of solvent, as the solvent with high boiling point in the TA remains in the part.

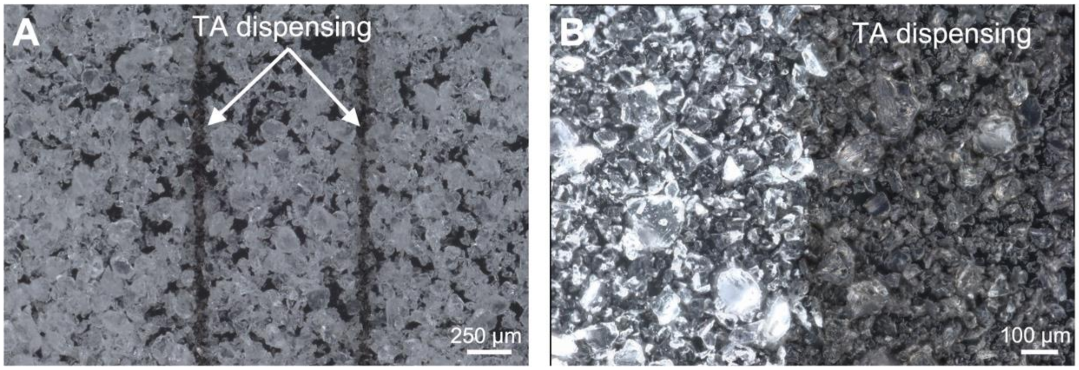

Figure S2. Optical microscope images of the TPU powder with TA dispensing (A) into two lines and (B) on the designated region.

Figure S3 shows the optical microscope images of the fused CNT/TPU layers obtained from the two CNT addition methods. The thin-layer samples were fabricated as follows. First, the neat TPU powder and premixed CNT/TPU powder were respectively loaded onto the glass substrates individually, with heat-resistant double layer tapes to fix a thin layer of powder. An air gun was used to remove redundant powder on the substrates. The TA was programed to dispense on the substrate loaded with a thin layer of the neat TPU powder (as the same volume of that dispensed during the printing process). Finally, the samples were then heat in an oven at 150 °C to fuse. Compared with the sample with TA dispensing, a few dark aeras with various size can be seen in the premixed CNT/TPU layer, confirming the higher tendency of CNT aggregation.

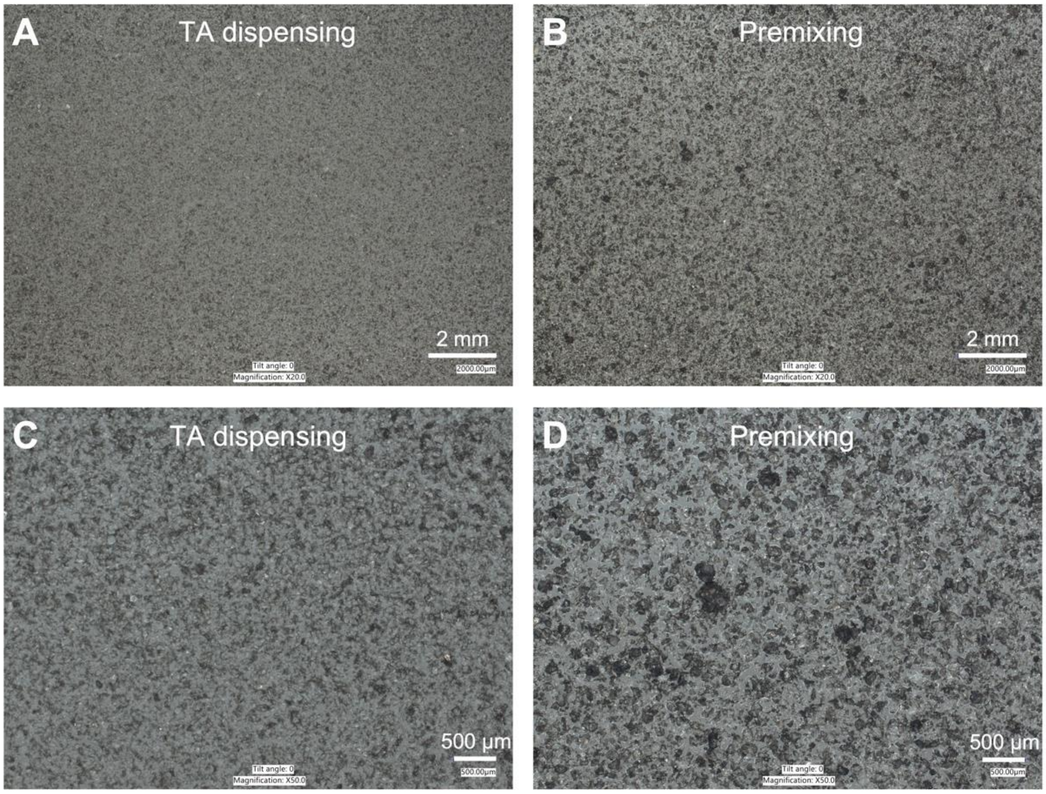

Figure S3. Optical microscope images of the fused CNT/TPU layers by (A) TA dispensing and (B) premixing.

Table S1. Physical properties of TA and fusing agent (FA).

Ink	Density (g/cm^3)	Viscosity (mPa·s)	Surface tension (mN/m)	*Z*
TA	1.043	3.4	42.7	7.9
FA	1.067	4.5	30.7	5.1

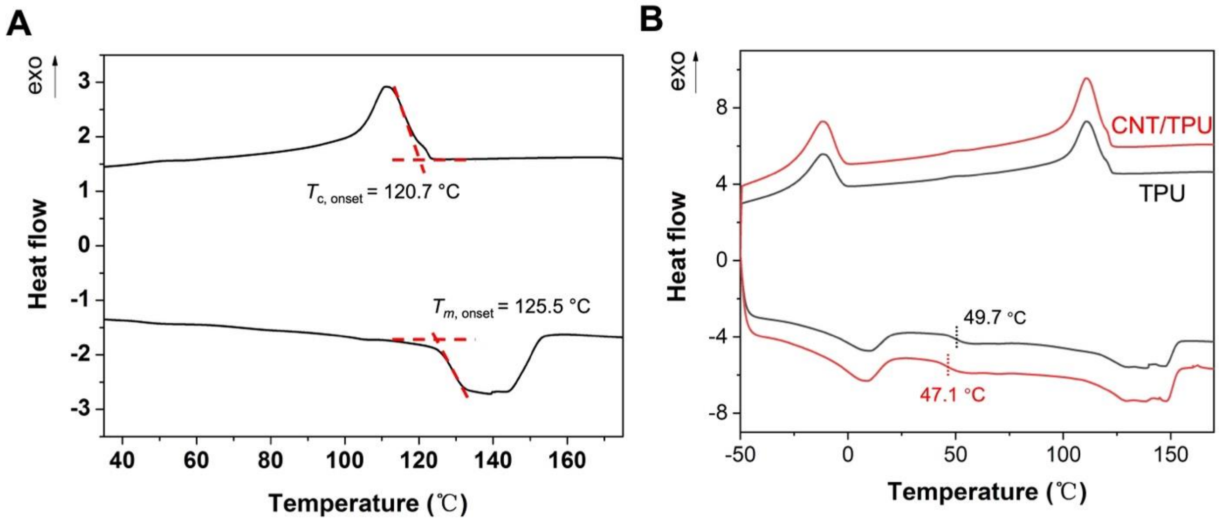

Figure S4. Differential scanning calorimetry curves of (A) the CNT/TPU part fabricated using TAs with 3 wt% CNT and neat TPU part fabricated using FA, and (B) TPU powder.

Table S2. Various effects on the tensile properties of the CNT/TPU parts using TAs and the TPU parts using FA.

Effects						UTS (MPa)	Young's modulus (MPa)	Elongation at break (%)	Toughness (MJ/m^3)
TA formulation			Printing parameters						
CNT content (wt%)	Solvent content (wt%)	Solvent type	Agent dispensing times	Layer thickness (μm)	Build orientation				
1	13	2-pyrrolidinone	1	80	*x*	10.3±0.5	78.3±3.2	442±41	35.2±4.9
2	13	2-pyrrolidinone	1	80	*x*	10.9±0.4	72.2±7.3	517±32	42.6±3.1
3	13	2-pyrrolidinone	1	80	*x*	11.8±1.1	80.1±7.2	541±44	48.3±4.4
4	13	2-pyrrolidinone	1	80	*x*	11.0±0.4	77.2±8.1	533±43	44.8±4.3
5	13	2-pyrrolidinone	1	80	*x*	10.3±0.6	73.2±7.3	483±52	38.2±5.6
3	20	2-pyrrolidinone	1	80	*x*	11.6±1.2	79.5±10.3	550±11	49.1±4.4
3	13	Glycerol	1	80	*x*	10.7±0.2	87.4±9.3	478±25	39.3±2.0
3	13	2-pyrrolidinone	2	80	*x*	8.9±0.4	81.4±2.3	351±49	23.1±5.0
3	13	2-pyrrolidinone	1	60	*x*	11.6±0.3	87.4±13.0	532±42	46.4±4.9
3	13	2-pyrrolidinone	1	100	*x*	11.3±0.6	76.9±11.5	512±34	43.8±5.7
3	13	2-pyrrolidinone	1	80	*y*	11.6±0.2	76.2±4.6	545±19	47.3±2.1
3	13	2-pyrrolidinone	1	80	*z*	11.2±0.3	70.0±10.4	421±24	36.0±2.9
0.5	30	2-pyrrolidinone	2	80	*x*	6.5±0.4	40.8±2.3	243±35	12.5±2.8
Control group (FA)			1	80	*x*	9.1±0.4	80.2±6.3	372±21	27.1±2.6
			1	80	*y*	8.4±0.4	74.0±5.7	283±43	19.1±3.7
			1	80	*z*	6.3±0.1	79.7±4.3	97.6±14.0	5.3±1.0

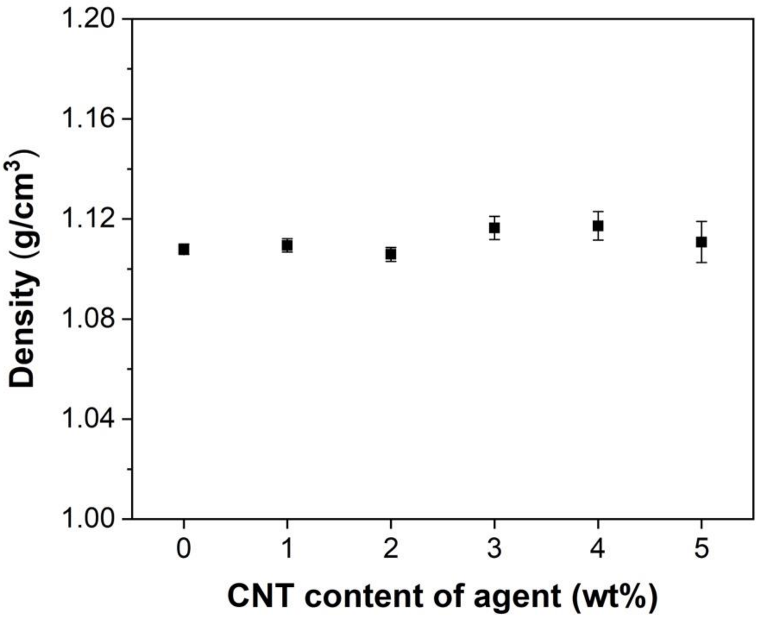

Figure S5. Density of the CNT/TPU parts fabricated using TAs with CNT content from 1 to 5 wt% and the neat TPU parts using FA.

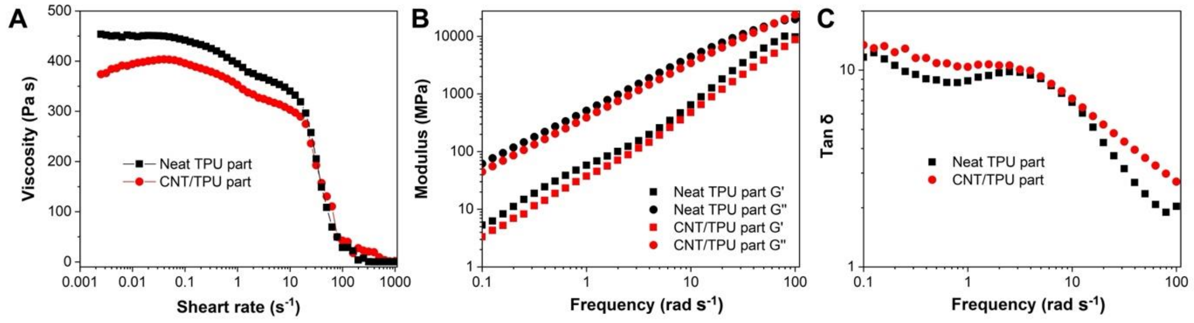

Figure S6. Rheological properties of the TPU part and CNT/TPU part at melting temperature. (A) Variations of the shear rate against viscosity. Variations of the frequency against (B) elastic modulus and viscous modulus (G′ and G′′, respectively) and (C) Tanδ.

Table S3. Average values of the mechanical properties in the *x*, *y*, *z* directions for the CNT/TPU and TPU parts.

Specimen	UTS (MPa)	Young's modulus (MPa)	Elongation at break (%)	Toughness (MJ/m^3)
CNT/TPU	11.5	75.4	502.3	43.9
TPU	7.9	78.0	251.0	17.2

To investigate the variation of hydrogen-bonding ratio after adding CNTs, Fourier-transform infrared spectroscopy (FTIR) was analyzed. Figure S7 shows the FTIR spectra of the CNT/TPU and TPU parts. The positions of the characteristic bands of the functional groups remained the same between the CNT/TPU and TPU parts, while the intensity ratios of some groups were different. The hydrogen bonds can be formed between N-H (3500–3200 cm^{-1}) and C=O groups (1800–1600 cm^{-1}), of which band intensities were increased in the CNT/TPU part as compared with those of the TPU part. The hydrogen-bonding index (HBI) that reflects the hydrogen-bonding ratio can be calculated by

$$HBI = \frac{A_{\mathrm{bonded}}}{A_{\mathrm{total}}} = \frac{A_{\mathrm{bonded}}}{A_{\mathrm{free}} + A_{\mathrm{bonded}}},$$

where A_{bonded}, A_{free}, and A_{total} are the integrated absorbance peaks of the hydrogen bonded groups, free groups, and total groups, respectively. Figure S8 shows the peak fitting results of the N-H and C=O groups of the TPU and CNT/TPU parts, and the HBI values of the groups are listed in Table S4. The CNT/TPU part possessed higher HBI values of the N-H (0.760) and C=O groups (0.548) than the TPU part, indicating the enhanced hydrogen-bonding interaction between the carboxylated CNTs and TPU.

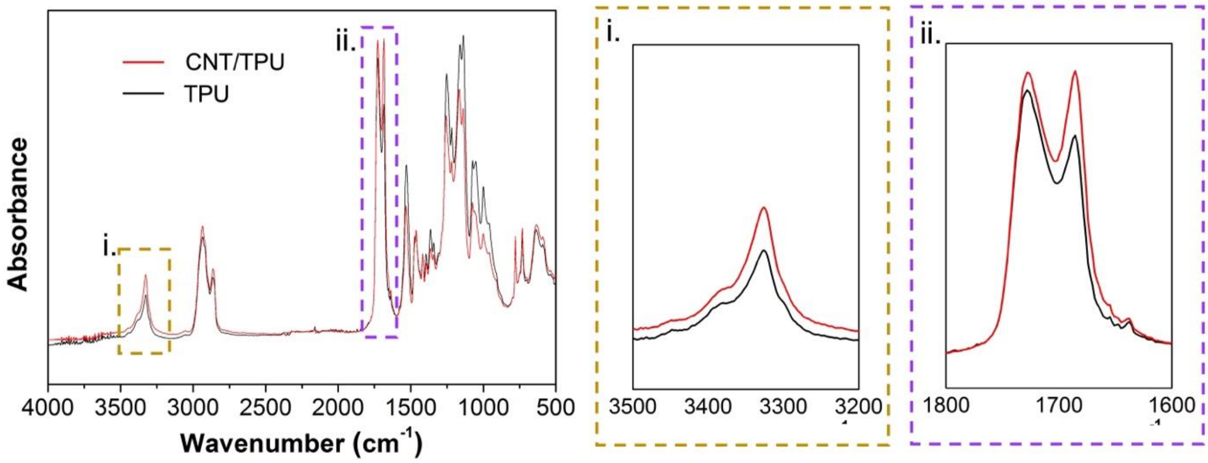

Figure S7. FTIR spectra of the CNT/TPU and TPU parts. Two characteristic bands were zoomed-in, corresponding to N-H and C=O groups.

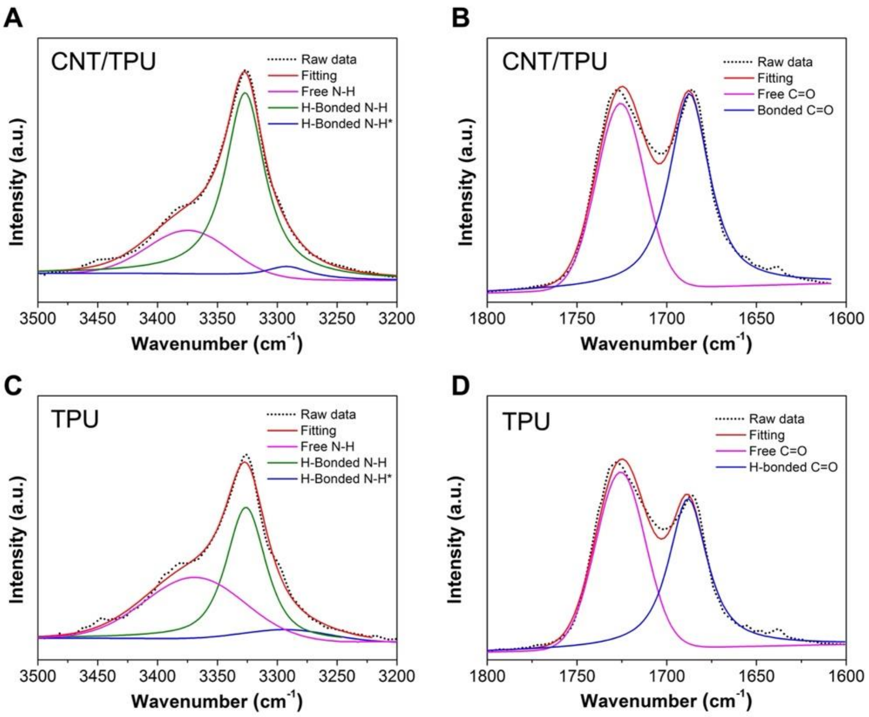

Figure S8. Peak fitting results of the N-H and C=O groups of the CNT/TPU and TPU parts.

Table S4. HBI values of the N-H and C=O groups of the CNT/TPU and TPU parts.

Part	N-H	C=O
CNT/TPU	0.760	0.548
TPU	0.567	0.480

Table S5. Mechanical properties of the recently reported CNT-filled TPU composites fabricated via FDM and casting.

	Part	Filler content (wt%)	UTS (MPa)	Variation * (%)	Elongation (%)	Variation # (%)	Ref.
FDM	Modified-MWCNT/TPU	3	21.7	+48.6	601	-14.6	[1]
FDM	MWCNT/TPU	4	~12	~+100	-	-	[2]
FDM	MWCNT/TPU	6	24.9	+199	~300	~-6.7	[3]
FDM	MWCNT/TPU	1	29.4	-8.6	644	+7.2	[4]
Casting	SWCNT/TPU	0.5	~55	~+15	~1100	~-3	[5]

Based on the estimated consumable cost in Table S6, the benefit of manufacturing toughened CNT/TPU part manufacturing can be further calculated. The cost of the CNT/TPU parts fabricated by the proposed process, $c_{CNT/TPU}$, is evaluated to 0.26 USD per cm^3 model. We take the toughness enhancement as an example to calculate the benefit of the CNT/TPU parts comparing with that of the normal TPU parts provided by HP.[6] The average increment of the

toughness of the CNT/TPU parts is 155%, compared with those of the neat TPU parts. To fulfill the same requirement of toughness, the consumption of the CNT/TPU part, $x_{CNT/TPU}$, is 1/(1+155%) times of the neat TPU parts (x_{TPU}=1). Hence, the benefit of CNT/TPU part manufacturing can be estimated as

$$y = \left(1 - \frac{x_{CNT/TPU} \cdot c_{CNT/TPU}}{x_{TPU} \cdot c_{TPU}}\right) \times 100\%,$$

and the benefit value, y, turns out as 63.6%.

Table S6. Consumable cost of the CNT/TPU parts applying 3 wt% TA and the neat TPU applying estimated by using HP MJF 4200 commercial printer.

Material	Volume per unit (L)	Numbers of consumed units per year	Price per SKU[&] (USD)	Price per year (USD)	Price per day (220/year) per cm^3 model (USD)	Price per cm^3 model (USD)
TPU	300	14	9,500.00[#]	133,000.00	604.55	0.15
TA	5	8	530.27	9,544.79	43.39	0.01
FA	5	8	1,500.00	27,000.00	122.73	0.03
DA	5	28	1,500.00	42,000.00	190.91	0.05
Spare parts	-	-	-	-	-	0.05
Total for CNT/TPU parts[*]	-	-	-	-	-	**0.26**
Total for TPU parts	-	-	-	-	-	**0.28**

[*] The calculation process was referred to the analysis from [6], and the costs of the CNT/TPU and TPU parts included the detailing agent (DA) part.
[&] SKU means stock keeping unit.
[#] Estimated price of TPU.

References

[1] D. Xiang, X. Zhang, Y. Li, E. Harkin-Jones, Y. Zheng, L. Wang, C. Zhao, P. Wang, *Compos. B: Eng.* **2019**, 176, 107250.
[2] J. F. Christ, N. Aliheidari, A. Ameli, P. Pötschke, *Mater. Des.* **2017**, 131, 394-401.
[3] L. Yang, X. Liu, Y. Xiao, Y. Zhang, G. Zhang, Y. Wang, *Adv. Mater. Technol.* **2023**, 8, 2201638.
[4] L. Tzounis, M. Petousis, S. Grammatikos, N. Vidakis, *Materials (Basel)* **2020**, 13, 2879.
[5] G. Zhu, F. Wang, L. Chen, C. Wang, Y. Xu, J. Chen, X. Chang, Y. Zhu, *Compos. Sci. Technol.* **2022**, 217, 109133.
[6] DigiFabster, HP's Multi Jet Fusion: How to Calculate Costs and Set Up Pricing. https://www.digifabster.com.
